# Disappearing Kilimanjaro snow—Are we the last generation to explore equatorial glacier biodiversity?

**DOI:** 10.1002/ece3.5327

**Published:** 2019-07-13

**Authors:** Krzysztof Zawierucha, Daniel H. Shain

**Affiliations:** ^1^ Department of Animal Taxonomy and Ecology, Faculty of Biology Adam Mickiewicz University Poznań Poland; ^2^ Biology Department Rutgers, The State University of New Jersey Camden New Jersey

**Keywords:** biodiversity lost, cold hotspots, equator, ex situ conservation, ice, psychrophiles

## Abstract

Glaciation accompanied our human ancestors in Africa throughout the Pleistocene. Regrettably, equatorial glaciers and snow are disappearing rapidly, and we are likely the last generation who will get to know these peculiar places. Despite the permanently harsh conditions of glacier/snow habitats, they support a remarkable diversity of life ranging from bacteria to animals. Numerous papers have been devoted to microbial communities and unique animals on polar glaciers and high mountains, but only two reports relate to glacial biodiversity in equatorial regions, which are destined to melt completely within the next few decades. Equatorial glaciers constitute “cold islands” in tropics, and discovering their diversity might shed light on the biogeography, dispersal, and history of psychrophiles. Thus, an opportunity to protect biota of equatorial glaciers hinges on ex situ conservation. It is timely and crucial that we should investigate the glacial biodiversity of the few remaining equatorial glaciers.

1

Glaciation accompanied our human ancestors in Africa throughout the Pleistocene and is considered a major factor that triggered human evolution (Gamble, Davies, Pettitt, & Richards, 2004; Maslin & Christensen, 2007). Although glacier cover in Africa strongly fluctuated over the last 100 thousands years (Downie, 1964; Kaser, Mölg, Cullen, Hardy, & Winkler, 2010; Mark & Osmaston, 2008; Rosqvist, 1990), regrettably, equatorial glaciers and snow are currently disappearing rapidly, and we are likely the last generation who may get to know these peculiar places (Thompson, Mosley‐Thompson, Davis, & Brecher, 2011). Despite the permanently harsh conditions of glacier/snow habitats, they support a remarkable diversity of life ranging from single‐cell microbes (e.g., Archae, algae, bacteria, fungi) to sophisticated, multicellular animals (e.g., ice worms, rotifers, tardigrades; Cook, Edwards, Takeuchi, & Irvine‐Fynn, 2016; Darcy, Gendron, Sommers, Porazinska, & Schmidt, 2018; Takeuchi, Nishiyama, & Li, 2010; Zawierucha, Buda, et al., 2018; Zawierucha, Kolicka, Takeuchi, & Kaczmarek, 2015). To date, only two reports have been published on glacial biodiversity on equatorial glaciers, and two on snow, habitats that are destined to disappear completely within the next few decades (Kuja, Makonde, Boga, Muigai, & Uetake, 2018; Nedbalová & Sklenár, 2008; Uetake et al., 2014; Zawierucha, Gąsiorek, et al., 2018). Will we make efforts to describe glacial equatorial biodiversity? Will we prolong the lives of equatorial glacier organisms in laboratories for further studies? What can we learn about these organisms and our coexistence with them?

Only a few places on Earth still host equatorial and near equatorial ice—Africa (Kenya, Tanzania, Uganda), New Guinea, and the largest equatorial ice cover, South America (Ecuador, Colombia; Figure 1). These glaciers are all located at least at 4,000 m a.s.l., higher than many tropical glaciers in South America or temperate glaciers in the Caucasus or Alps (Veettil & Kamp, 2019). Considering the hundreds of studies devoted to microbial communities and invertebrates on glaciers and snow in the Arctic, Antarctic, Third Pole (Hindu Kush–Karakoram–Himalayan), and other high mountain regions (Cook et al., 2016; Hodson et al., 2008; Hotaling, Hood, & Hamilton, 2017; Kaczmarek, Jakubowska, Celewicz‐Gołdyn, & Zawierucha, 2016; Zawierucha et al., 2015), the scarce knowledge collected from tropical icy islands seems alarming, from the perspective of biodiversity protection. Equatorial and tropical glaciers (e.g., in central Asia, South America) are considered a source of freshwater maintaining downstream animals and plants assemblages (Bosson, Huss, & Osipova, 2019; Milner et al., 2017; Veettil & Kamp, 2019). Also equatorial, glacier‐fed streams present unique hydraulic patterns when compared to temperate regions, and taxon richness in glacier‐fed streams of the Ecological Reserve of Antisana (Ecuador) will be significantly reduced following glacier shrinking (Cauvy‐Fraunié et al., 2013). But these equatorial glaciers are also unique ecosystems and may be sources of nutrients and first migrants in cold water streams (Bagshaw et al., 2013; Dresch et al., 2019; Pessi et al., 2018).

**Figure 1:**
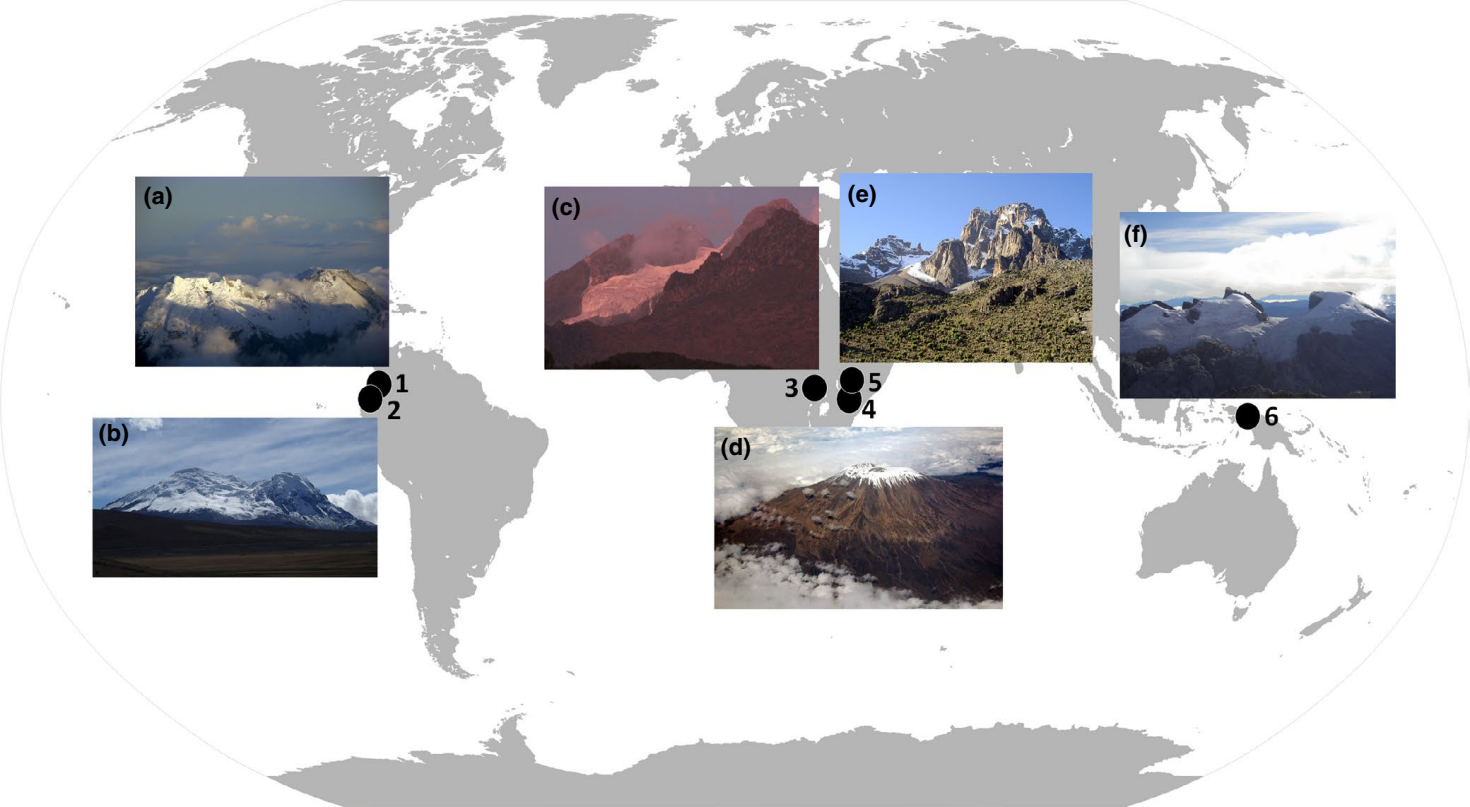
Map presenting equatorial areas still hosting glaciers. 1. Colombia, 2. Ecuador, 3. Uganda, 4. Tanzania, 5. Kenya, 6. New Guinea. (a) Nevado del Huila (https://commons.wikimedia.org/wiki/File:Volcan_Huila_9-12-2008_(1).jpg, Martin Roca [CC BY 3.0]), (b) Antisana (https://upload.wikimedia.org/wikipedia/commons/8/8d/Antisana_im_September_2018.jpg, Stefan Weigel [CC BY‐SA 4.0]), (c) Rwenzori (Courtesy Jun Uetake], (d) Kilimanjaro (https://upload.wikimedia.org/wikipedia/commons/c/c6/Kilimanjaro_%28paulshaffner%29.jpg, Paul Shaffner [CC BY 2.0], (e) Mount Kenya (https://commons.wikimedia.org/wiki/File:Pt_Thomson_Batian_Nelion_Mt_Kenya.JPG#file, [CC BY‐SA 3.0]), (f) Puncak Jaya (https://upload.wikimedia.org/wikipedia/commons/5/5d/Sumantri_%28center%29_with_Ngga_Pulu_%28right%29_from_Carstensz_Summit_by_Christian_Stangl_flickr.jpg, Christian Stangl [CC BY‐SA 2.0]

Historically, glaciers have been considered sophisticated parts of our natural landscape, space for explorers, recreation, and a symbol of the wilderness (Carey, 2007). Currently, however, glaciers and ice sheets are considered natural hazards as contributors to sea‐level rise and an economic threat due to shrinking of frozen freshwater reservoirs (Beniston, Stoffel, & Hill, 2011). For biologists, glaciers were once considered unproductive and sterile, but in the last few decades, this view has been fully reversed with discoveries of extremophilic glacier organisms across the domains of life (Edwards et al., 2014; Shain et al., 2016; Takeuchi, Kohshima, Yoshimura, Sekto, & Fujita, 2000; Zawierucha et al., 2015). Although glacial organisms (e.g., algae, invertebrates) were first observed in the 19th century (Drygalski, 1897), glaciers as an important ecological habitat remained forgotten since that time (Hodson et al., 2008). Abiotic views of glaciers as simply water in a frozen state have evolved into peculiar biomes, differing from polar and high mountain ecosystems by unique organism assemblages, topographic features, and climate (Anesio & Laybourn‐Parry, 2012). Although glaciers have been a particular focus of polar biologists, ecologists, and biochemists over the past two decades (Bagshaw et al., 2013; Cook et al., 2016; Dial, Ganey, & Skiles, 2018; Takeuchi et al., 2000), we emphasize that equatorial glacier habitats, biodiversity, and ecosystems remain mostly unexplored (Uetake et al., 2014; Zawierucha, Gąsiorek, et al., 2018). We identified marginal knowledge on equatorial glacier biodiversity using search engines such as Scopus, Web of Science, and literature published herein. We supported our investigation using Google scholar. Using a set of keywords related to glacial ecosystems (Table 1), we revealed that this knowledge is almost nonexistent in comparison with tropical, mountain, and polar glaciers. Even though some data on equatorial ice are hidden under the term tropical (Kuja et al., 2018; Zawierucha, Gąsiorek et al., 2018), still knowledge of equatorial glaciers is limited. Certainly, as shown in Table 1, a number of published studies correlate with the area of glaciation. Even though equatorial glaciers are small (i.e., playing marginal roles in global cycles in comparison with polar ice caps, perhabs influencing small scientfic interests), due to their high speed of melting and their unique location, they should be studied urgently. Nonetheless, it seems their existence was and still is forgotten. Jan Carstenszoon, a Dutch explorer who visited New Guinea in 1623, reported on the existence of Puncak Jaya snow to citizens of the old continent, which was ridiculed by those who were skeptical about glaciers in the tropics. Since then, equatorial glaciers have been mostly neglected in the biological sciences and are mostly within the scope of glaciologists (Kaser et al., 2010; Milner et al., 2017; Thompson, Brecher, Mosley‐Thompson, Hardy, & Mark, 2009; Thompson et al., 2011).

**Table 1:** Results of search in scientific browsers Scopus and WoS (Web of Science) using titles, abstracts, keywords, and topic of papers in relation to ecosystems on glaciers (Accessed on 09.05.2019)

Browser	Keywords				
	Equator, glacier	Tropic, glacier	Alpine^a^, glacier	Arctic, glacier	Antarctic, glacier
Scopus	89	276	2,692	4,194	3,362
WoS	63	167	2,941	2,241	2,546

	Equator, glacier, cryoconite^b^	Tropic, glacier, cryoconite	Alpine, glacier, cryoconite	Arctic, glacier, cryoconite	Antarctic, glacier, cryoconite
Scopus	0	0	41	84	35
WoS	0	0	66	112	51

	Equator, glacier, biodiversity	Tropic, glacier, biodiversity	Alpine, glacier, biodiversity	Arctic, glacier, biodiversity	Antarctic, glacier, biodiversity
Scopus	1	8	102	65	58
WoS	3	4	139	68	58

	Equator, glacier, supraglacial	Tropic, glacier, supraglacial	Alpine, glacier, supraglacial	Arctic, glacier, supraglacial	Antarctic, glacier, supraglacial
Scopus	1	2	81	151	44
WoS	1	0	91	93	54

a Alpine refers to the type of polar and high mountain glaciers.

b Cryoconite is dark sediment on the glacier surface (from ancient Greek “*kryos*”—cold and “*konis*”—dust, Nordenskiöld (1875)) comprising mineral particulate matter of local and remote origin, including organic compounds, bacteria, algae, fungi, which in turn support protozoans and invertebrates. Cryoconite is a basic element in the functioning of supraglacial ecosystems (Cook et al., 2016; Hodson et al., 2008; Takeuchi, 2002; Takeuchi et al., 2010).

Scientific investigation has produced two studies on equatorial glacial biology, and both are devoted to organisms on one Ugandan glacier (Figure 1, Figure 3 A‐B). Uetake et al. (2014) reported a novel biogenic aggregation of mainly protonemal moss gemmae and protonema (called GMGA—glacier moss gemmae aggregation). Importantly, these aggregations are home to numerous microorganisms, and the inevitatble loss these glaciers (located at Rwenzori Mountains) will lead to the loss of this unique habitat. Subsequently, Zawierucha, Gąsiorek, et al. (2018) discovered rotifers and tardigrades in GMGA and described a new tardigrade species, *Adropion afroglacialis* (and another identified to the genus level, Figure 2), emphasizing the need for equatorial glacial biodiversity conservation. Additionally, Kuja et al. (2018) and Nedbálova & Sklenár (2008) studied snow packs on Mount Kenya and Ecuador respectively, emphasizing the extinction threat of cold‐adapted microbes. However, as on glaciers of polar, alpine and third pole regions, scientists should expect more species of metazoans on and in the vicinity of equatorial glaciers, together with novel species of primary producers and bacteria. Glaciers worldwide as supraglacial ecosystems encompass habitats like streams, lakes, cryoconite holes (water‐filled reservoirs on glacier surfaces), weathering crusts, glacier mice (moss balls), dirt cones, and tills (Cook et al., 2016; Coulson & Midgley, 2012; Franzetti et al., 2017; Hodson et al., 2008; Zawierucha, Buda, et al., 2018; Figure 3). Parts of equatorial glaciers covered by perennial snow also may constitute viable habitats based on recently discovered distinct and isolated biogeographic communities on snow in various parts of the world (Kuja et al., 2018; Lutz et al., 2016; Onuma et al., 2017). Moreover, glacial ecosystems include not only supraglacial environments, but also englacial and subglacial channels which, despite much research in polar regions, remains enigmatic, yet inhabited by diverse microbial communities (Achberger et al., 2017). In equatorial zones, these habitats are more speculatory than empirically recognized. To date, only GMGA and snow have been described, while other potential habitats remain undiscovered.

**Figure 2:**
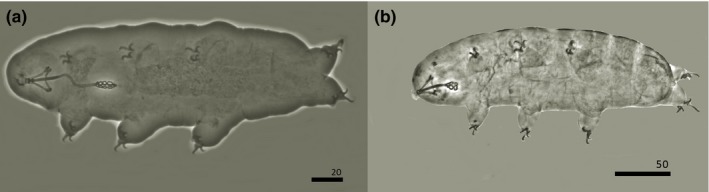
Water bears (Tardigrada) found on disappearing Ugandan glacier. (a) *Adropion afroglacialis* Zawierucha, Gąsiorek, et al., 2018, (b) *Hypsibius* sp. (scale bar given in micrometers)

**Figure 3:**
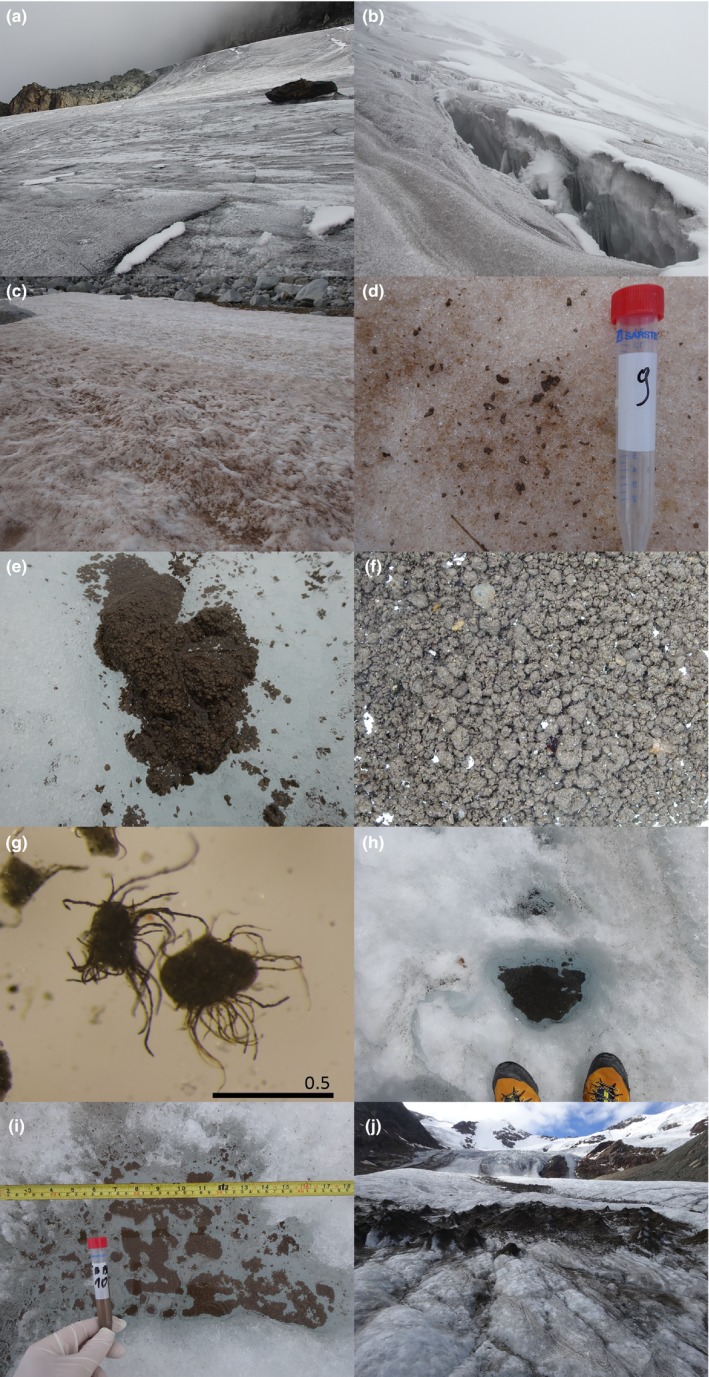
A, B. Dark matter (cryoconite) on glacier surface in Uganda (courtesy Elżbieta Wiejaczka), mineral grains , most likely along with various microorganisms, e.g., ice algae, cyanobacteria (Cook et al., 2016; Gerdel & Drouet, 1960). Analogues to potential snow and supraglacial features on equatorial glaciers. C. Red snow on snow patches in the Pyrenees during summer. Effect of algae (*Chlamydomonas* sp.) blooming; D. Red snow with first aggregations (granules) formed by microorganisms; E, F. Cryoconite granules, bioreactors formed due to interaction of cyanobacteria (glacial ecosystem engineers) and other biotic and abiotic components on ice (Langford, Hodson, Banwart, & Bøggild, 2010; Takeuchi et al., 2010; Uetake et al., 2016), G. Cryoconite granule overgrown by cyanobacteria (scale bar in millimeter); H, I. Cryoconite holes with cryoconite granules on bottom (H ‐ in the Alps, I – in Spitsbergen (small holes connected into one puddle)); J. Dirt cones in the Alps

Knowledge on psychrophiles and their habitats is important in both basic and applied sciences (Hulatt, Berecz, Egeland, Wijffels, & Kiron, 2017; Segawa et al., 2018; Singh, Hanada, Singh, & Tsuda, 2014; Stibal et al., 2017). Equatorial glaciers constitute, “cold islands” for life in tropical regions and discovering their diversity might shed light on the biogeography of psychrophiles, their metabolism, dispersal, and history. A particular difference between equatorial and polar glaciers is that the former follows both seasonal and daily cycles. Due to high altitude, their surface is irradiated intensively, they represent different geological settings, and mostly they are not directly surrounded by high mountains (like glaciers in Svalbard or the Alps), which decreases the delivery of local mineral and organic material on the glacier surface. The glaciated areas in Colombia and Ecuador belong to the inner tropics, with a year‐round precipitation pattern that lacks any seasonality (Veettil & Kamp, 2019). These climatic and geological differences likely shape different microbial assemblages in comparison with polar or alpine glaciers. Biodiversity on equatorial glaciers may even be important in explaining biological processes from the distant past. Currently, water reservoirs on large ice sheets are considered as potential analogue for refugia during the period of Snowball Earth (Hawes, Jungblut, Matys, & Summons, 2018; Hoffman, 2016). In turn, small equatorial glaciers may constitute analogues for the end of Snowball Earth, and the processes and fate of organisms on these glaciers may reflect the end of glaciation during global warming, shedding light on their adaptation to a rapidly changing environment.

**Figure 4:**
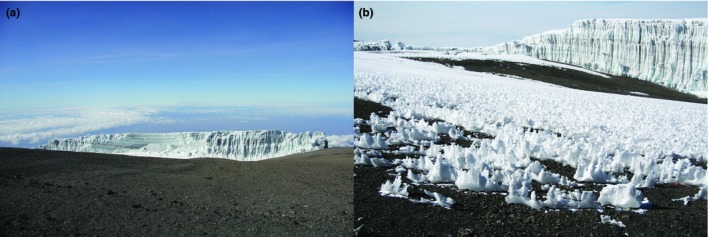
Glaciers on Kilimanjaro. Pictures were taken on A. February 2018, and B. September 2010. Courtesy Elżbieta Wiejaczka

Unfortunately, the story of equatorial glaciers is quickly coming to an end before its scientific inquiry has really begun—important scientific data are literally melting away. Melting ice and snow packs in Antropocene is not only triggered by temperature, but in tropical regions is more complex due to the effects of deforestation, influencing rain, and atmospheric humidity (Basantes‐Serrano et al., 2016; Kaser et al., 2010; Klein & Kincaid, 2006; Mölg, Rott, Kaser, Fischer & Cullen, 2006; Thompson et al., 2009). Most of glaciers on Kilimanjaro (Figure 4 A‐B) will most likely disappear within 25 years, while small glaciers in New Guinea near the Puncak Jaya peak will last only a few more decades (Thompson et al., 2009, 2011); the Ugandan glacier probably will be gone in <5 years (Mölg et al., 2006; Taylor, Mileham, Tindimugaya, Majugu, Muwanga, & Nakileza, 2006). Fairing slightly better are South American glaciers, although those proximal to the Equator are melting very quickly (Thompson et al., 2011). For example, the glaciers on Antisana volcano in Ecuador, a few degrees south of the Equator, will survive <50 years (Basantes‐Serrano et al., 2016). Strong fluctuations in equatorial ice cover occurred during the late Pleistocene and early Holocene (Downie, 1964; Kaser et al., 2010; Mark & Osmaston, 2008; Thompson et al., 2009), but current global shrinking of ice is incontestable. Because small, fragmented ecosystems are more influenced by changes in temperature, rainfall, and humidity compared with large contiguous ecosystems, trends in natural fragmentation of such ecosystems are an indicator of their vulnerability to global changes (Krauss et al., 2010; Mantyka‐Pringle, Martin, & Rhodes, 2012). Equatorial glaciers and snow patches, due to their size and location, seem to be the most sensitive climatic indicators and may serve as a symbol of disappearing and unknown ecosystems in an era of changes triggered by human activity (deforestation, greenhouse gas production, releasing of soot and particulates decreasing ice albedo etc.).

Bosson et al. (2019) analyzed 19,000 glaciers worldwide (including those in equatorial regions) stating that glaciers may play the same function as umbrella, keystone, and flagship species in biological species conservation. Such divisions may also be applied to equatorial ice. As umbrella species, maintaining equatorial glaciers will allow the conservation of other features threatened by melting like disappearing glacier habitats, changes of the landscape or local climate (Carey, 2007). Unfortunately, many studies indicate that the existence of equatorial glaciers is almost over, and we cannot protect these glaciers before mass extinctions. As keystone species, these glaciers support the existence of cold streams, which are home for unique assemblages of plants and animals, and also plants and animals on glaciers (Milner et al., 2017; Zawierucha, Gąsiorek, et al., 2018). Finally, as flagship species, these glaciers increase awareness about the loss of ice ecosystems, the most visible changes of natural ecosystems on Earth and their unknown biodiversity at the Equator; thus, equatorial glaciers serve as a last endangered endemic species.

The prediction of a changing global climate mobilized world leaders in 1992 to reverse these warming trends, leading to an international environmental treaty “United Nations Framework Convention on Climate Change (UNFCCC)” with an objective to stabilize greenhouse gases associated with the widespread loss of biodiversity. Since then, however, no comprehensive attempts have been made to investigate or protect biodiversity on tropical and equatorial glaciers, which are at high risk of being lost forever, along with their unique biota. Since the announcement of the so‐called convention on biodiversity, four volumes of “Global Biodiversity Outlook” (GBO) have been published, which provides a summary of the status of biodiversity and an analysis of efforts taken by the scientific community toward biodiversity conservation. One of the focal areas in the second version of GBO (Secretariat of the Convention on Biological Diversity & UNEP World Conservation Monitoring Centre, 2006) was reducing loss of the components shaping biodiversity, including (a) habitats, (b) species, and (c) genetic diversity. In terms of small equatorial glaciers and snow patches, ecological restoration of habitats is almost impossible, and certainly, we will not artificially recover equatorial glacial diversity and function. Thus, it is critically important to grasp what we can. Glaciers worldwide contain important information about our past climate and environments within the layers of ice. These layers also store biological information, and along with microorganisms from the surface and bedrock of glaciers, they should be stored for future generations in biological banks. GBO 1 highlights the opportunity of ex situ conservation. Such examples include seed banks (in glacial communities the equivalent are ice core banks) as well as organizations which store biological materials for analysis. Taking into account that the species extincton rate is 100–1,000 higher than before human existence (Pimm, Russel, Gitleman, & Brooks, 1995), it is undisputable that propagation of representative species and cultures of tractable glacial organisms for longer term analyses is crucial and should be made available for scientists worldwide.

Considering that marine and terrestrial equatorial regions are a biodiversity hotspot (Tapia‐Armijos, Homeier, Espinosa, Leuschner, & de la Cruz, 2015), an extrapolation can reasonably be made to equatorial glaciers. Do we forgot that species on Earth are awaiting discovery, and that many undescribed species inhabiting this biodiversity hotspot are becoming extinct? We cannot forget that the recognition of systems and diveristy on our planet is a characteristic of natural human curiosity and expansion of our knowledge base; it is timely and crucial that we should investigate the glacial dynamics, ecology, and biological diversity of the few remaining equatorial glaciers before the opportunity is lost. It is very important and highly appropriate that mankind should study hypothetical ice‐based life on other planets and moons (Martin & McMinn, 2018), but it is alarming that we forget about the last glaciers which we can explore in our home, on Earth.

## CONFLICT OF INTEREST

None declared.

## **AUTHOR** **CONTRIBUTIONS**

KZ conceived the study, wrote the manuscript, and revised and edited the manuscript. DS contributed to the text and revisions.

## Data Availability

Data sharing is not applicable to this article as no new data were created or analyzed in this study.
